# Supplementary material for: Using a latent Hawkes process for epidemiological modelling
Source: PLoS One. 2023 Mar 1;18(3):e0281370. doi: 10.1371/journal.pone.0281370 (PMC9977047; doi:10.1371/journal.pone.0281370)
Supplement: S1 Appendix — (PDF) [file pone.0281370.s001.pdf]

# 1 Appendix

**S1** To assess the performance of our algorithm on synthetic data, we find the Average Absolute Error (AAE) of the computed estimates :

$$\mathrm{AAE}(\hat{\lambda}^N) = \frac{1}{N_t}\sum_{i=1}^{N_t} |\hat{\lambda}^N(x_i) - \lambda^N(x_i)|$$

$$\mathrm{AAE}(\hat{R}) = \frac{1}{16}\sum_{i=1}^{16} |\hat{R}_i - R_i|$$

and the Root Mean Square Error (RMSE):

$$\mathrm{RMSE}(\hat{\lambda}^N) = \sqrt{\frac{1}{N_t}\sum_{i=1}^{N_t}\left(\hat{\lambda}^N(x_i) - \lambda^N(x_i)\right)^2}$$

$$\mathrm{RMSE}(\hat{R}) = \sqrt{\frac{1}{16}\sum_{i=1}^{16}(\hat{R}_i - R_i)^2},$$

where $N_t$ is the number of test time points $x_i$ randomly chosen in the time-horizon we consider, $\hat{\lambda}^N(x_i)$ and $\lambda^N(x_i)$ the estimated via posterior median and true intensity at time $x_i$, $\hat{R}_i$ and $R_i$ the estimated via posterior median and true reproduction number in the $i_{th}$ week.

**S2** We conduct a sensitivity analysis in the mean of GI and IP using the real cases in the local authority Ashford (19/12/2021 - 9/4/2022) [28]. Figure 13 shows the estimated reproduction number, the estimated weekly latent cases and the estimated latent intensity varying the mean of GI and keeping fixed its SD at 1.8 days and the mean and SD of IP at 8.8 days and 4.4 days, respectively. The analysis shows that the weekly latent cases and the estimated latent intensity are not sensitive to the changes in the mean of GI. On the other side, we notice intense differences in the estimated reproduction number related to week 11. The weekly observed cases show a sharp rise between weeks 10 and 13, corresponding to an increase of latent cases in former weeks, given the maximum delay of 21 days between the reported and actual infection times. In a predetermined interval, smaller GI leads to higher infections compared to higher GI explaining the smaller estimated reproduction number with GI at 3.7 days and the higher estimated reproduction number at 6.7 days related to week 11 to catch the increase of latent cases that period.

Figure 14 show the estimated reproduction number, the estimated weekly latent cases and the estimated latent intensity varying the mean of IP and keeping fixed its SD at 4.4 days and the mean and SD of GI at 6.7 days and 1.8 days, respectively. The transition kernels of observed cases with mean at 5.8 days and 6.8 days illustrate that an event is most likely to be observed five and three days after the actual infection time, respectively. The observed differences in the estimates are ought to the various values of delay between observed and actual infection times.

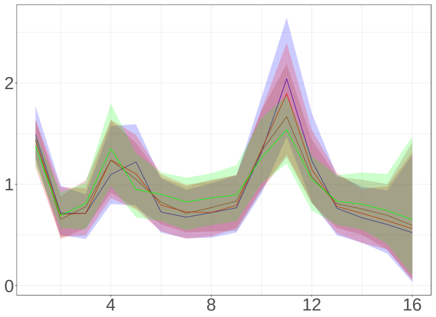


**(a)** Reproduction number

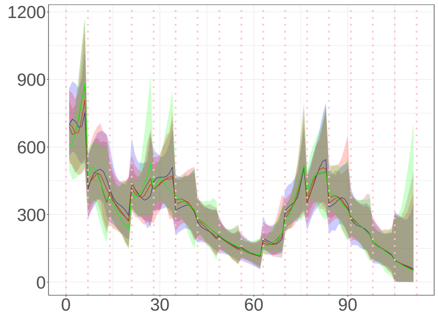


**(b)** Weekly latent cases

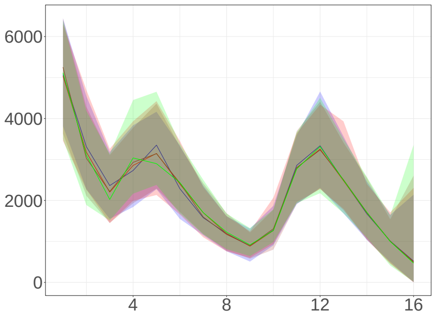


**(c)** Intensity of latent cases

**Figure 13. The reproduction number, the intensity of latent cases and the weekly latent cases over various values of the mean of GI (8.7 days (blue line); 5.7 days (red line); 4.7 days (brown line); 3.7 days (green line)) plotted against time.** The time interval between two successive pink vertical dashed lines corresponds to a week.

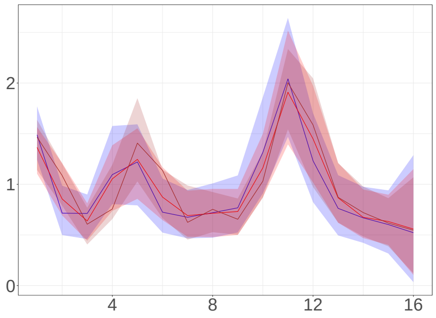


**(a)** Reproduction number

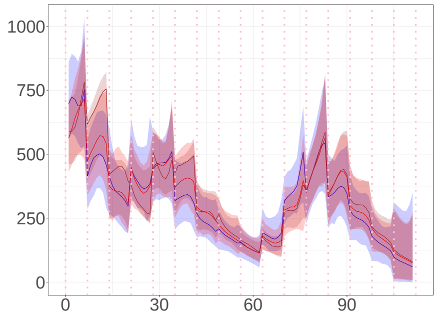


**(b)** Weekly latent cases

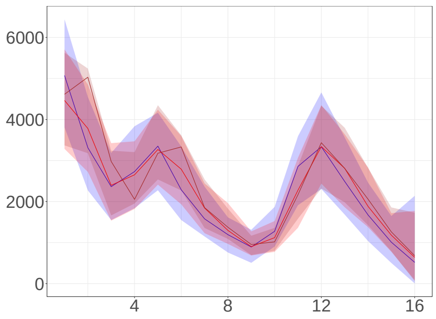


**(c)** Intensity of latent cases

**Figure 14. The reproduction number, the intensity of latent cases and the weekly latent cases over various values of the mean of IP (8.8 days (blue line); 6.8 days (red line); 5.8 days (brown line)) plotted against time.** The time interval between two successive pink vertical dashed lines corresponds to a week.
